# Maternal preconception circulating blood biomarker mixtures, child behavioural symptom scores and the potential mediating role of neonatal brain microstructure: the S-PRESTO cohort

**DOI:** 10.1038/s41398-023-02332-6

**Published:** 2023-02-03

**Authors:** Jian Huang, Ai Peng Tan, Evelyn Law, Keith M. Godfrey, Anqi Qiu, Lourdes Mary Daniel, Marielle Fortier, Kok Hian Tan, Jerry Kok Yen Chan, David Cameron-Smith, Yap Seng Chong, Shiao-Yng Chan, Johan G. Eriksson, Michael J. Meaney, Jonathan Huang

**Affiliations:** 1 Singapore Institute for Clinical Sciences (SICS), Agency for Science, Technology and Research (A*STAR), Singapore, Singapore; 2 Department of Diagnostic Radiology, Yong Loo Lin School of Medicine, National University of Singapore (NUS), Singapore, Singapore; 3 Department of Diagnostic Imaging, National University Health System, Singapore, Singapore; 4 Department of Paediatrics, Yong Loo Lin School of Medicine, National University of Singapore, Singapore, Singapore; 5 Department of Paediatrics, Khoo Teck Puat-National University Children’s Medical Institute, National University Hospital, Singapore, Singapore; 6 MRC Lifecourse Epidemiology Centre and NIHR Southampton Biomedical Research Centre, University of Southampton & University Hospital Southampton NHS Foundation Trust, Southampton, UK; 7 Department of Biomedical Engineering, National University of Singapore, Singapore, Singapore; 8 The N.1 Institute for Health, National University of Singapore, Singapore, Singapore; 9 NUS (Suzhou) Research Institute, National University of Singapore, Singapore, China; 10 Institute of Data Science, National University of Singapore, Singapore, Singapore; 11 Department of Biomedical Engineering, Johns Hopkins University, Baltimore, USA; 12 Department of Child Development, KK Women’s and Children’s Hospital, Singapore, Singapore; 13 Department of Diagnostic & Interventional Imaging, KK Women’s and Children’s Hospital, Singapore, Singapore; 14 Department of Maternal Fetal Medicine, KK Women’s and Children’s Hospital, Singapore, Singapore; 15 KK Research Centre, KK Women’s and Children’s Hospital, Singapore, Singapore; 16 College of Health, Medicine and Wellbeing, The University of Newcastle, Newcastle, Australia; 17 Department of Obstetrics & Gynaecology, National University Health System, Singapore, Singapore; 18 Yong Loo Lin School of Medicine, Human Potential Translational Research Programme, National University of Singapore, Singapore, Singapore; 19 Department of Obstetrics & Gynaecology, Yong Loo Lin School of Medicine, National University of Singapore, Singapore, Singapore; 20 Department of general practice and primary health care, University of Helsinki, Helsinki, Finland; 21 Folkhälsan Research Center, Helsinki, Finland; 22 Department of Psychiatry, Faculty of Medicine and Ludmer Centre for Neuroinformatics and Mental Health, Douglas Hospital Research Centre, McGill University, Montreal, Canada; 23 Centre for Quantitative Medicine, Duke-NUS Medical School, Singapore, Singapore

**Keywords:** Predictive markers, Human behaviour, Psychiatric disorders

## Abstract

Human brain development starts in the embryonic period. Maternal preconception nutrition and nutrient availability to the embryo may influence brain development at this critical period following conception and early cellular differentiation, thereby affecting offspring neurodevelopmental and behavioural disorder risk. However, studying this is challenging due to difficulties in characterizing preconception nutritional status and few studies have objective neurodevelopmental imaging measures in children. We investigated the associations of maternal preconception circulating blood nutrient-related biomarker mixtures (~15 weeks before conception) with child behavioural symptoms (Child Behaviour Checklist (CBCL), aged 3 years) within the Singapore Preconception Study of Long-Term Maternal and Child Outcomes (S-PRESTO) study. The CBCL preschool form evaluates child behaviours based on syndrome scales and Diagnostic and Statistical Manual of Mental Disorders (DSM) oriented scales. These scales consist of internalizing problems, externalizing problems, anxiety problems, pervasive developmental problems, oppositional defiant, etc. We applied data-driven clustering and a method for modelling mixtures (Bayesian kernel machine regression, BKMR) to account for complex, non-linear dependencies between 67 biomarkers. We used effect decomposition analyses to explore the potential mediating role of neonatal (week 1) brain microstructure, specifically orientation dispersion indices (ODI) of 49 cortical and subcortical grey matter regions. We found that higher levels of a nutrient cluster including thiamine, thiamine monophosphate (TMP), pyridoxal phosphate, pyridoxic acid, and pyridoxal were associated with a higher CBCL score for internalizing problems (posterior inclusion probability (PIP) = 0.768). Specifically, thiamine independently influenced CBCL (Conditional PIP = 0.775). Higher maternal preconception thiamine level was also associated with a lower right subthalamic nucleus ODI (*P*-value = 0.01) while a lower right subthalamic nucleus ODI was associated with higher CBCL scores for multiple domains (*P*-value < 0.05). One potential mechanism is the suboptimal metabolism of free thiamine to active vitamin B1, but additional follow-up and replication studies in other cohorts are needed.

## Introduction

The most common behavioural problems in childhood and adolescence include anxiety, aggressive behaviour, attention deficit hyperactive disorder (ADHD), and pervasive developmental problems including autism spectrum disorder (ASD) [1, 2]. Such behavioural problems can be generally classified as internalizing disorders such as anxiety and externalizing disorders such as aggressive behaviour [1]. A previous study found 12.2% and 4.9% of Singaporean primary school children (6–12 years) had experienced internalizing and externalizing problems, respectively [3], which is comparable to global prevalence estimates [4]. Child and adolescent behavioural problems have far-reaching impacts on adult life, including poorer academic outcomes, work incapacity, drug use, and other addictive behaviours [2, 5].

Child and adolescent behavioural problems are influenced by various factors, including genetics [6, 7], maternal education [8], maternal depression [9], parenting [1], and adverse socio-economic environment [1]. Maternal nutritional status before pregnancy has been linked with pregnancy and child health outcomes [10], and nutritional supplements are commonly recommended to promote better pregnancy outcomes [11]. However, it is unclear whether and how preconception nutrient status may influence foetal and offspring brain development and ultimately child behaviours. Mechanisms underlying neurodevelopmental disorders involve variations in brain anatomy, functioning, and connectivity [12, 13]. Magnetic resonance imaging (MRI) studies suggest differential brain structural characteristics, detectable even as early as the first two years of life, may underlie neurodevelopmental disorders [14–16] Importantly, human brain development such as dendritic morphology starts in the embryonic period [17, 18], making it essential to understand the role of modifiable environmental exposures during this critical period. In particular, maternal preconception nutrition and fetal nutrient availability may influence brain development around the time of conception and early cellular differentiation [19], thereby affecting offspring neurodevelopmental outcomes such as behavioural disorders. However, due to the challenges of prospectively following women who are not yet pregnant, few studies have investigated the associations of preconception biomarkers, alone or in combination, with child neurodevelopmental outcomes [20]. Importantly, existing studies finding associations have not been able to evaluate the role of potential mechanisms such as via changes in offspring brain microstructure. Consequently, many findings are circumstantial, e.g., correlations to pregnancy or postnatal diet or maternal underlying health status, and do not necessarily propose potential causal exposures or periods for intervention.

Notably, past studies have relied on self-reported preconception supplement use or only considered objective biomarkers during pregnancy, which may be influenced by changes to diet and metabolism during pregnancy or be too late to capture the early peri-conceptional period. Moreover, studies have focused on biomarkers in isolation without respecting their interdependencies due to dietary patterns and/or related metabolic pathways. To address this, we leveraged a prospective, pregnancy and child cohort to investigate the relationships between preconception nutrition-related biomarkers and child behavioural symptoms using novel approaches to account for biomarker clustering and interdependence. To strengthen inference, we further explored the role of neonatal brain microstructure in mediating such associations.

## Materials and methods

### Data Source

This study was conducted within the Singapore PREconception Study of long-Term maternal and child Outcomes (S-PRESTO) cohort [21]. In brief, between February 2015 and October 2017, S-PRESTO recruited 1032 non-pregnant women aged 18 to 45 years (mean = 31, standard deviation (SD) = 3.7) of Chinese, Malay or Indian ethnicity who intended to conceive and deliver in Singapore. Participants were followed for up to 3 preconception visits and censored at 12 months if they did not conceive. During the preconception visits, baseline characteristics such as educational levels/attainment, household income, and medical history were assessed using interviewer-administered questionnaires, and self-administered mood questionnaires were also completed. Importantly, fasting blood samples were collected at the first preconception visit.

**Fig. 1:**
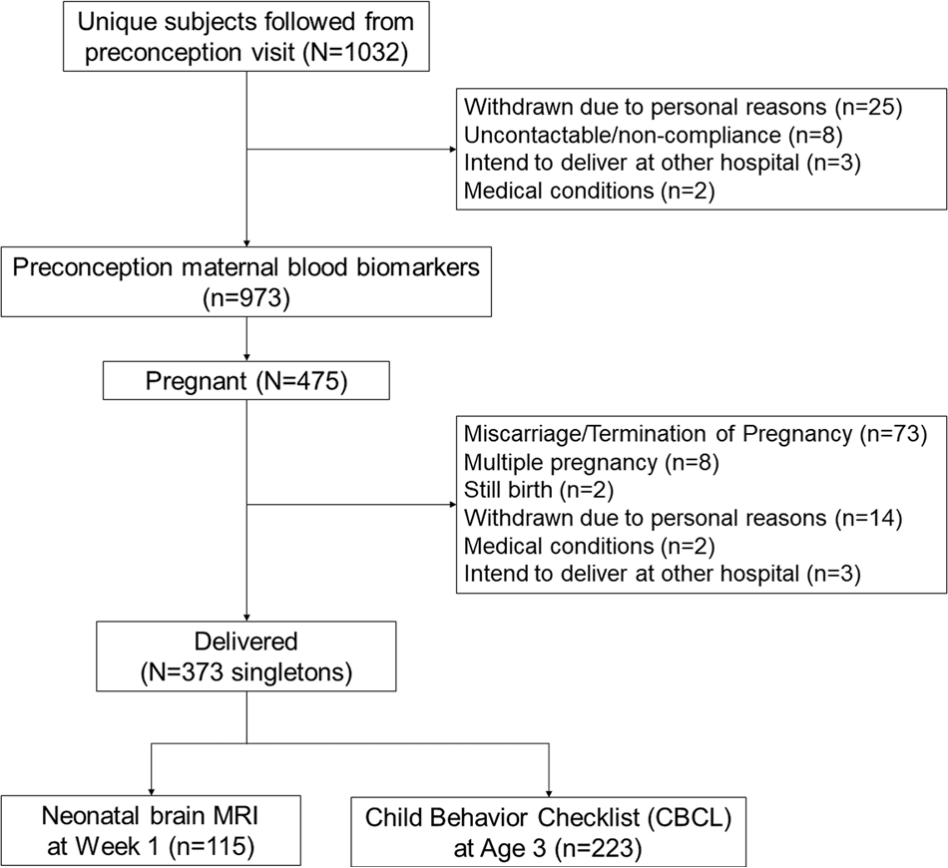
Flowchart for study samples from the Singapore Preconception Study of Long-Term Maternal and Child Outcomes (S-PRESTO) prospective study. Each box in the flowchart indicates the sample sizes for data collected at various time points and the number of participants being excluded.

A total of 475 women successfully conceived, among whom 373 singleton children were born in the cohort. Women and their children were followed up after delivery to collect data on standardized anthropometric measurements, brain magnetic resonance imaging, and neurodevelopmental outcomes. Figure 1 shows the flowchart and sample sizes for maternal blood biomarkers, neonatal brain MRI, and child behaviour assessment. Written informed consent was obtained from all participants. Ethical approval was obtained from the SingHealth Centralised Institutional Review Board (reference 2014/692/D) [21].

#### Maternal biomarkers

Circulating maternal levels of 72 biomarkers were measured using fasting blood samples collected during the first preconception visit (*n* = 973). Seventy of the biomarkers were measured using a well-validated mass spectrometry platform (BEVITAL platforms B, C, D, and H, https://bevital.no/) in EDTA plasma samples, which quantifies amino acids, vitamins, acylcarnitines and other metabolites. The remaining two biomarkers, cobalamin (vitamins B12) and folate (vitamin B9), were measured using a chemiluminescent immunoassay (Beckman platform) in EDTA plasma samples.

Zero values were reported for more than 80% of the women for five preconception biomarkers: nicotinic acid, pyridoxine, vitamin D2, cotinine, and hydroxycotinine. Nicotinic acid, pyridoxine, and vitamin D2 are forms of vitamin B3, vitamin B6 and vitamin D, respectively, which exist in other forms that are measured in the platform used. Cotinine and hydroxycotinine are metabolites of nicotine and low levels of these metabolites are due to a low smoking rate among women in Singapore and our cohort [22]. We excluded these biomarkers from our analysis. The remaining 67 biomarkers included 46 metabolites, 12 micronutrients, and 9 essential amino acids (EAAs). Raw values were natural log-transformed after adding one and results are reported per SD unit of the natural log-transformed data.

We identified 11 clusters for the 67 preconception biomarkers using K-means clustering, which minimizes the within-cluster sum of squares (Supplementary Table 1) [23]. Since metabolites and micronutrients may represent different underlying constructs, for example, long-term metabolism and short-term dietary patterns, respectively, we performed a sensitivity analysis accounting for the two categories of biomarkers separately: (1) metabolites; (2) micronutrients/EAAs. We identified eight clusters for preconception metabolites and seven clusters for preconception micronutrients and EAAs (Supplementary Table 1).

#### Child behaviour checklist

The parent-reported CBCL preschool form was administered at age 3 years and responses were received from 223 children (mean = 3.1 years, SD = 0.1 years). The CBCL preschool form evaluates child behaviours based on syndrome scales and Diagnostic and Statistical Manual of Mental Disorders (DSM) oriented scales. Syndrome scales consist of internalizing problems (emotionally reactive, anxious/depressed, somatic complaints, withdrawn) and externalizing problems (attention problems and aggressive behaviour), sleeping problems, and total problems. DSM-oriented scales consist of ADHD, affective problems, anxiety problems, pervasive developmental problems, and oppositional defiant. Raw scores were natural log-transformed after adding one, and results are reported per SD unit of the natural log-transformed scores. A higher CBCL score indicates greater problems on each scale.

#### Neonatal brain magnetic resonance imaging

Neonatal brain MRI was performed in 115 infants within the first week after birth using a 3-Tesla scanner (Magnetom Skyra, Siemens Healthineers, Erlangen, Germany). Multishell diffusion-weighted sequence was acquired. A total of 109 infants with mean absolute motion smaller than 3 mm (average mean absolute motion of 0.95 mm and an interquartile range of 0.69–1.03 mm) were included in our analysis. Diffusion images were preprocessed using FMRIB’s Diffusion Toolbox, FSL v6.0.4, and fitted to the Neurite Orientation Dispersion and Density Imaging (NODDI) model using the NODDI MATLAB Toolbox v1.05 [24]. Neuroimaging parameters are presented in Supplementary Table 2. NODDI uses a multi-segmental model of the cellular and extracellular compartments of each voxel, and provides a more biologically specific representation of brain development [25]. In our analysis, we used the orientation dispersion index (ODI) estimated from the NODDI model to indicate the angular distribution of neurites (range from 0 to 1). ODIs of 49 cortical and subcortical grey matter regions were extracted using segmentation masks from the developing human connectome project (dHCP, v1.1) pipeline [26]. A higher value of ODI indicates a higher degree of dendritic complexity [24]. Dendritic spines are the main gateway of excitatory synaptic transmission in the brain. Hence, a higher degree of dendritic complexity will facilitate information transfer between brain regions [27]. Conversely, a lower ODI, which indicates a lower degree of dendritic complexity, may signify impairment of information transfer between brain regions, a probable mechanism underlying neurodevelopmental disorders such as ASD [18].

### Statistical analysis

#### Preconception maternal biomarkers and offspring CBCL scores

Among the mother-child dyads with both biomarker and CBCL data, we excluded two dyads of mixed ethnicity. Given that biomarkers do not act independently, we applied a mixture method (Bayesian kernel machine regression, BKMR) to account for the complex interaction between biomarkers under investigation in this study [28]. In brief, BKMR uses a kernel machine representation to model a high-dimensional exposure-outcome response surface by assuming that individuals with similar exposure profiles have similar health risks [28]. To reduce the number of model inputs amongst highly correlated exposures, BKMR incorporates Bayesian variable selection. Variable selection was implemented using a Markov chain Monte Carlo (MCMC) algorithm and posterior inclusion probabilities (PIPs) were estimated using a Bayesian model-averaging method. In this study, we considered each of the 15 CBCL scores separately and performed the main analysis for Biomarkers→CBCL, and sensitivity analyses for Metabolites→CBCL and Micronutrients/EAAs→CBCL. For each analysis, we performed 60,000 MCMC iterations with 12 independent chains with a burn-in of 30,000 MCMC iterations. Within each iteration, we assumed that each biomarker within a cluster was equally likely to be included in the model and only one biomarker from a cluster was selected into the model at a time. The variable selection parameter was estimated as the probability density function from the prior distribution. We tested gamma, uniform, and inverse uniform prior distributions. Effective sample sizes for the MCMC sampler were small and potential scale reduction factors were large for both gamma and uniform distributions, indicating MCMC samples were highly correlated and the estimates were not robust. Therefore, we chose inverse uniform distribution for variable selection parameter estimation. Given that correlated biomarkers and/or biomarker clusters may act on the same biological pathways, we applied a hierarchical variable selection approach which first estimates the Posterior Inclusion Probability (PIP) for each biomarker cluster (Cluster PIP), and then the PIPs among biomarkers within each cluster, given that the cluster was selected into the model (Conditional PIP). In this way, Cluster PIPs and Conditional PIPs indicate the proportions of all models in which the particular clusters or the biomarker within a cluster were being selected. We reported suggestive associations with a threshold of Cluster PIP > 0.5 and Conditional PIP > 0.5 and highlighted more plausible associations using a threshold of Cluster PIP > 0.75 and Conditional PIP > 0.5. We also filtered the candidate associations with MCMC effective sample size greater than 100 and a potential scale reduction factor smaller than 1.1 to ensure the reliability of our analysis. A potential scale reduction factor of 1.1 indicates that increasing the number of iterations to infinity can reduce the interval width of the estimate by 10% [29].

For interpretability, we used the models to estimate effects as the difference in the mean outcome (CBCL measure) when a single exposure was set to a level corresponding to the 75th percentile (observed in the study) as compared to when it is set to the 25th percentile, while all other model exposures were set to their observed median values [28]. As a comparison, we also performed standard multivariable linear regression. We performed linear regression using samples with complete data and using inverse probability weighting (IPW) to account for potential biases associated with loss to follow-up. The missingness model for IPW was based on mother’s highest educational level, household income, maternal age, and maternal preconception body-mass index, given that distributions of these covariates differed between the sub-sample with both biomarker and CBCL data and those without such data (Supplementary Table 3).

To investigate whether ethnicity and socio-economic status (SES) may modify these associations, we performed BKMR and linear regressions stratified by (1) maternal ethnicity (Chinese vs Malay; Indian was not included due to a small sample size); (2) mother’s highest educational level (University degree or above v.s. Polytechnic or below); and (3) household income (Higher household income group (6–10 decile) vs Lower household income group (1–6 decile)).

#### Preconception maternal biomarker and neonatal brain MRI measures

We further explored the role of neonatal brain MRI measures in the candidate biomarker-CBCL associations. Since the number of biomarkers was greater than the sample size available for this analysis, we were unable to fit BKMR models for this analysis. We investigated biomarker-ODI associations and ODI-CBCL associations using linear regression. We first investigated the associations of thiamine and thiamine monophosphate (TMP) with ODI of 49 cortical and subcortical grey matter regions. Thiamine and TMP were selected because they were the most promising findings in the main biomarker-CBCL analysis. The findings with a raw *p*-value smaller than 0.05 in the biomarker-ODI association analysis were selected for the ODI-CBCL association analysis. We presented both raw *p*-values and false discovery rate (FDR) in each stage of the analysis. In addition, we performed regression-based causal effect decomposition analyses [30] to evaluate the potential mediating role of MRI measures in the thiamine-CBCL associations. This approach computes the pure and total (including interaction) direct effects, and pure and total (including mediated-interaction) indirect effects [30]. This allows us to explore both mediation and interaction simultaneously. Because this exploratory analysis was underpowered due to the nature of mediation analyses and the necessity of having complete data on biomarkers, ODI, and CBCL, we focused on the 95% CI and present uncorrected p-values. We estimated the proportion mediated as total natural indirect effect divided by total effect, and the corresponding 95% CI was estimated using the delta method [31].

#### Covariates

To increase the precision of estimates and take account of potential confounders, we adjusted for covariates associated with the exposure and/or the outcome, including child sex, age at CBCL assessment, maternal ethnicity, mother’s highest education, household income, maternal age at preconception visit, nulliparity, maternal preconception body-mass index (BMI), and gestational age at birth in Model 1. Household income was categorized into deciles based on resident employed households in Singapore, ranging from the lowest (Below S$1622 per month) to the highest (S$16,601 or above per month) household income group. We used the 10 levels of household income as a continuous variable in our non-stratified analyses. In the SES-stratified analysis, we stratified household income into a lower household income group (levels 1–6) and a higher household income group (levels 6–10). Household income level 6 was included in both subgroups otherwise we would not be able to perform BKMR in at least one subgroup due to the small sample size. Among mothers who had had livebirths, household income was classified as level 6 (S$7424–S$9082) in 25%, levels 1–6 (Below S$9083 per month) in 60%, and levels 6–10 (S$7424 or above per month) in 65%. Although preconception nutritional status may influence maternal mental health, a bidirectional relationship between nutrition and mental health and a mediating pathway of maternal depressive symptoms inversely affecting child cognitive function via unhealthy nutrition have been reported [32]. It has been more clearly shown that more depressed and anxious mothers differentially report child behavioural problems [33, 34]. Therefore, we additionally adjusted for maternal preconception mental health score in Model 2. Maternal preconception mental health score was derived from Beck Depression Inventory (BDI-2), Edinburgh Postnatal Depression Scale (EPDS), and State-Trait Anxiety Inventory (STAI) using principal component analysis (PCA). The first principal component (PC) from PCA explained 98% of the variance across all three inventories and was used to indicate maternal mental health. A higher score indicates worse mental health.

#### Statistical analysis software

All analyses were performed using R 4.1.3. BKMR analyses were performed using the *bkmr* package and regression-based causal effect decomposition analyses were performed using the *regmedint* package. All tests were two-sided tests.

## Results

### Descriptive analysis

Of the 373 women who successfully conceived and remained in our cohort at the time of delivery, 322 had data on preconception biomarkers, 109 had data from neonatal MRI scans, and 223 had CBCL assessment at age 3 years. In our main analysis, 196 mother-child dyads had available data on both preconception biomarkers and CBCL assessment. Table 1 showed that mother-child dyads with and without preconception biomarkers and CBCL assessment were similar with respect to most characteristics. We also compared the participants with biomarker and CBCL data for the analysis to those who successfully conceived but were lost to follow-up (Supplementary Table 3). Those who had available data on biomarkers and CBCL assessment reported a higher household income level. Compared to those initially recruited but did not conceive within 12 months or lost to follow-up, those with available data for this analysis were younger and had a higher educational level, higher household income, a lower BMI, and better preconception mental health status (Supplementary Table 3). These factors were adjusted for in the downstream association analyses. Supplementary Table 4 shows that the medians and interquartile ranges for biomarkers under investigation were similar between the full sample and the sub-sample available for BKMR analysis and mediation analysis.

**Table 1:** Comparisons of characteristics between the participants with and without biomarker and CBCL data (among those who had given livebirth).

Covariate	Category	Livebirth without biomarker and CBCL data				Livebirth with biomarker and CBCL data				*P*-value^a^
		*N*	%	Mean	SD	*N*	%	Mean	SD	
Overall		177				196				
Child sex	Girl	79	44.6%			89	45.4%			1
	Boy	93	52.5%			107	54.6%			
Maternal ethnicity	Chinese	139	78.5%			146	74.5%			0.129
	Indian	10	5.6%			16	8.2%			
	Malay	21	11.9%			32	16.3%			
	Mixed ethnicity	7	4.0%			2	1.0%			
Mother’s highest educational level	University degree or above	125	70.6%			143	73.0%			0.699
	Polytechnic or below	52	29.4%			53	27.0%			
Household income^b^		145		5.8	2.1	163		6.3	2.1	0.037
Maternal age at preconception visit (years)		177		30.2	3.2	196		30.7	3.3	0.142
Estimated number of weeks before gestation		166		21.9	17.2	185		15.6	15.9	4.2 × 10^–4^
Maternal preconception BMI (kg/m^2^)		175		23.2	4.5	194		22.8	4.0	0.402
Nulliparity	No	64	36.2%			76	38.8%			0.710
	Yes	112	63.3%			120	61.2%			
Gestational age at birth (weeks)		177		38.9	1.3	196		38.8	1.4	0.333
Maternal mental health score		123		54.3	14.2	136		52.8	13.7	0.400

*BMI* body-mass index, *CBCL* Child Behaviour Checklist, *SD* standard deviation.

^a^*P*-values for differences comparing the participants who had given livebirth with biomarker and CBCL data and those who had given livebirth but without biomarker and CBCL data were estimated from chi-squared test for categorical characteristics and from *t*-test for continuous characteristics.

^b^Household income was categorized into levels 1–10 indicating the lowest to the highest household income group based on the SPRESTO cohort. We used the 10 levels of household income as a continuous variable in our analyses.

### Maternal biomarkers and offspring CBCL scores

**Fig. 2:**
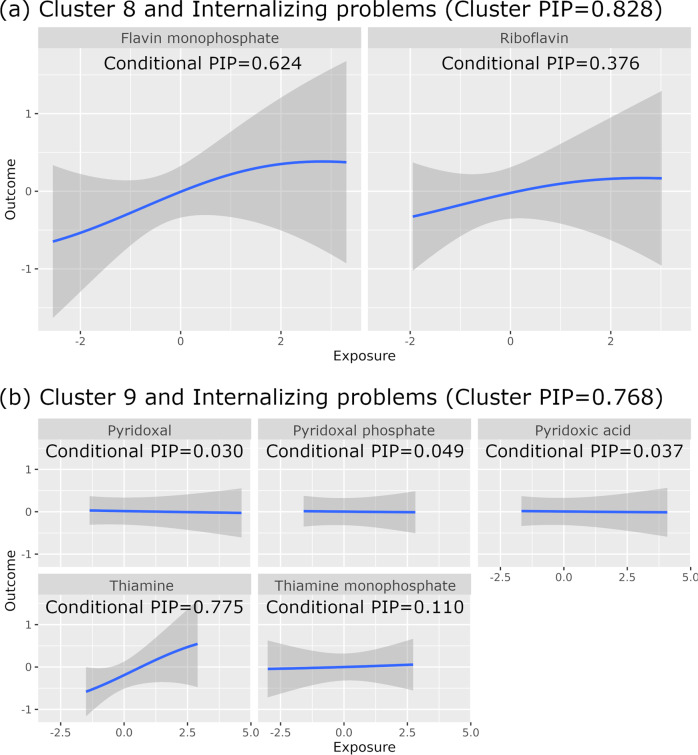
Exposure-outcome relationships using Bayesian kernel machine regression (BKMR) for biomarker clusters and Child Behaviour Checklist (CBCL) scores (Model 2, *N* = 109). Biomarker levels and CBCL scores were in standard deviation unit. Grey areas indicate 95% credible interval (PIP: posterior inclusion probability). **a** The association of Biomarker Cluster 8 with internalizing problems. **b** The association of Biomarker Cluster 9 with internalizing problems.

Table 2 shows the associations with a Cluster PIP greater than 0.75 in our fully adjusted model (Model 2). Flavin monophosphate had the highest Conditional PIP for the positive association of Biomarker Cluster 8 (riboflavin and flavin monophosphate) with internalizing problems (Cluster PIP = 0.828, Conditional PIP = 0.624) and thiamine had the highest Conditional PIP for the positive association of Biomarker Cluster 9 (thiamine, TMP, pyridoxal phosphate, pyridoxic acid, and pyridoxal) with internalizing problems (Cluster PIP = 0.768, Conditional PIP = 0.775). The above Cluster PIPs indicate that Biomarker Clusters 8 and 9 were selected in 82.8% and 76.8% of the MCMC iterations, respectively. The above Conditional PIPs indicate that flavin monophosphate was selected in 62.4% of the MCMC iterations where Biomarker Cluster 8 was selected, and thiamine was selected in 77.5% of the MCMC iterations where Biomarker Cluster 9 was selected. Figure 2 shows linear positive associations of preconception flavin monophosphate and thiamine with internalizing problems. This is consistent in the direction of effect with the finding from linear regression with complete data (Table 2) as well as linear regression with IPW (Supplementary Table 5). We estimated from the linear regression with complete data that per SD higher log-transformed flavin monophosphate level (equivalent to 36% higher in absolute flavin monophosphate level) was associated with 0.177 SD higher in the CBCL score for internalizing problems (equivalent to 16% higher in raw CBCL score). Similarly, per SD higher log-transformed thiamine level (equivalent to 40% higher in absolute thiamine level) was associated with 0.312 SD higher in the CBCL score for internalizing problems (equivalent to 28% higher in raw CBCL score). Suggestive associations with a Cluster PIP greater than 0.5 (but smaller than 0.75) and a Conditional PIP <0.5 for one biomarker in the corresponding cluster are reported in Supplementary Table 5. Biomarker Cluster 8 was positively associated with total problems (Cluster PIP = 0.631), somatic complaints (Cluster PIP = 0.583), and anxiety problems (Cluster PIP = 0.557). Biomarker Cluster 9 was positively associated with somatic complaints (Cluster PIP = 0.741), anxiety/depression (Cluster PIP = 0.587), affective problems (Cluster PIP = 0.529), and total problems (Cluster PIP = 0.529). Biomarker Cluster 6 (dimethylglycine, choline, methyl methacrylate (MMA)) was inversely associated with internalizing problems (Cluster PIP = 0.619), anxiety/depression (Cluster PIP = 0.604), anxiety problems (Cluster PIP = 0.544), and total problems (Cluster PIP = 0.519).

**Table 2:** Associations between maternal preconception circulating biomarker levels and Child Behaviour Checklist (CBCL) scores (Model 2).

Cluster	Biomarker	CBCL outcome	BKMR^a^ (*N* = 109)				Linear regression (complete data, *N* = 117)			
			Cluster PIP	Conditional PIP	Estimate^b^	SE	Beta	SE	*P*-value	95% CI
8	Flavin monophosphate	Internalizing	0.828	0.624	0.189	0.243	0.177	0.102	0.084	−0.024 to 0.377
8	Riboflavin	Internalizing	0.828	0.376	0.115	0.217	0.182	0.093	0.049	0.001 to 0.364
9	Thiamine	Internalizing	0.768	0.775	0.220	0.231	0.312	0.089	5.0 × 10^–4^	0.136 to 0.487
9	Thiamine monophosphate	Internalizing	0.768	0.110	0.025	0.101	0.241	0.095	0.011	0.054 to 0.428
9	Pyridoxal phosphate	Internalizing	0.768	0.049	0.004	0.033	0.149	0.095	0.118	−0.038 to 0.336
9	Pyridoxic acid	Internalizing	0.768	0.037	0.003	0.028	0.146	0.090	0.105	−0.030 to 0.323
9	Pyridoxal	Internalizing	0.768	0.030	0.000	0.015	0.096	0.084	0.256	−0.070 to 0.261

*BKMR* Bayesian kernel machine regression, *CBCL* Child Behaviour Checklist, *CI* confidence interval, *PIP* posterior inclusion probability, *Rhat* potential scale reduction factor, *SE* standard error.

^a^BKMR analysis for each CBCL outcome was performed by simultaneously accounting for 67 biomarkers (11 clusters). This table presents the associations that meet the following criteria: (1) Cluster PIP is greater than 0.75; (2) Conditional PIP for one biomarker in the corresponding cluster is greater than 0.5; (3) MCMC effective sample sizes are greater than 100 for all biomarkers in the cluster; and (4) Rhat (potential scale reduction factor) are smaller than 1.1 for all biomarkers in the cluster. Model 2 adjusted for child sex, age at CBCL assessment, maternal ethnicity, mother’s highest education, household income, maternal age at preconception visit, nulliparity, maternal preconception body-mass index, gestational age at birth, and maternal preconception mental health score.

^b^Effect estimates from BKMR indicate the difference in the mean outcome when a single exposure is fixed at its 75th percentile as compared to when it is fixed at its 25th percentile when all of the other exposures are fixed at their median value.

The sensitivity analysis considering only metabolites also showed a suggestive association between preconception TMP and internalizing problems (Cluster PIP = 0.696, Conditional PIP = 0.683; Supplementary Table 6), while the sensitivity analysis considering only micronutrients and EAAs showed a suggestive association between preconception thiamine and internalizing problems (Cluster PIP = 0.719, Conditional PIP = 0.870; Supplementary Table 7). In addition, associations were found for both TMP and thiamine with pervasive developmental problems (TMP: Cluster PIP = 0.893, Conditional PIP = 0.833, Supplementary Table 6; thiamine: Cluster PIP = 0.946, Conditional PIP = 0.958, Supplementary Table 7).

Supplementary Table 8 shows the results of the ethnicity- and SES-stratified analyses for Biomarker Clusters 6, 8, and 9 with internalizing problems as these were key findings in the main analysis. Ethnicity- and SES-stratified analyses using linear regression showed that thiamine and TMP were associated with internalizing problems in the higher household income group (β_thiamine_ = 0.361, P_thiamine_ = 0.001; β_TMP_ = 0.327, P_TMP_ = 0.014 in Model 2; Supplementary Table 8) but not in the lower household income group (β_thiamine_ = 0.172, P_thiamine_ = 0.166; β_TMP_ = 0.082, P_TMP_ = 0.355 in Model 2; Supplementary Table 8). Ethnicity- and SES-stratified BKMR accounting for all biomarkers simultaneously was not performed due to the small sample size adjusting for Model 2 covariates. Nevertheless, in Model 1, Biomarker Cluster 9 was associated with internalizing problems in the higher household income group (Cluster PIP = 0.818, Conditional PIP for TMP = 0.893; Supplementary Table 8 and Supplementary Fig. 1) but not in the lower household income group (Cluster PIP = 0.339, Conditional PIP for TMP = 0.306; Supplementary Table 8 and Supplementary Fig. 1). Supplementary Tables 9–12 show the additional results for ethnicity- and SES-stratified analyses using the BKMR model.

### Preconception maternal biomarker and MRI measures

A higher preconception thiamine level was nominally inversely associated with neonatal right subthalamic nucleus ODI (*N* = 56, *P*-value = 0.010, FDR = 0.972, Supplementary Table 13, Supplementary Figs. 2 and 3). Neonatal right subthalamic nucleus ODI was inversely associated with multiple CBCL scores (*N* = 37, Supplementary Table 14), including attention problems (*P*-value = 0.001, FDR = 0.008), ADHD (*P*-value = 0.005, FDR = 0.041), and externalizing problems (*P*-value = 0.012, FDR = 0.062). Causal mediation analyses showed that preconception thiamine was nominally associated with CBCL total problems (*N* = 63, *β* = 0.551, *P* = 0.038, 95% CI 0.030–1.072 for total effect). However, pure (*P* = 0.856) and total (*P* = 0.909) natural indirect effects via neonatal right subthalamic nucleus ODI for this association were not significant (Supplementary Table 15 and Supplementary Fig. 4). We estimated that the proportion mediated via neonatal right subthalamic nucleus ODI was 1.8% (95% CI −29% to 33%). No major differences have been found between pure and total natural indirect effects, suggesting mediated interaction was not likely.

## Discussion

In this study, for the first time, we have identified associations of maternal preconception circulating biomarkers with child behavioural symptoms scores at age 3 years. In a fully adjusted model, we found that Biomarker Cluster 9 (thiamine, TMP, pyridoxal phosphate, pyridoxic acid, and pyridoxal) was positively associated with internalizing problems and thiamine was driving the cluster association. While investigating metabolites separately from micronutrients and EAAs, associations of thiamine and TMP with internalizing problems remained, albeit with lower Cluster PIPs. Both thiamine and TMP were also consistently associated with pervasive developmental problems in the sensitivity analyses. In general, we found evidence of a link between maternal preconception thiamine-pathway-related biomarkers and child behaviours. In the SES-stratified analysis, these associations remained only in the high household income group.

Our findings of higher preconception free thiamine and TMP levels associated with internalizing problems were heavily influenced by higher scores on the somatic complaints and anxiety/depression scales, and pervasive developmental problems, which are mainly characterized by delays in the development of socialization and communication skills. This is not consistent with clinical knowledge, where both prenatal and infantile thiamine deficiency has been widely reported to be associated with undesirable child development outcomes, including impairment in language and communication skills [35–37]. Thiamine deficiency is typically viewed as a health problem due to poor diets in low- and middle-income countries and excessive alcohol consumption in high-income countries [38], which have a low prevalence in our population and preconception cohort. However, the true risk of insufficiency and/or suboptimal status of thiamine in women of reproductive age is not well understood [39]. Nevertheless, high-carbohydrate diets and consumption of sugar-sweetened beverages increase the demand for thiamine diphosphate (TDP), which is the active form of vitamin B1 (accounting for ~80% of the total thiamine content) and an essential cofactor for carbohydrate metabolism [40, 41]. In blood, TDP predominantly presents in erythrocytes, while free thiamine and TMP are found primarily in plasma [41]. Biomarkers were measured using plasma samples in our cohort and only free thiamine and TMP were available for this study. Compared to healthy Japanese women of the same age range [42], preconception levels of free thiamine and TMP were lower in our cohort (Supplementary Table 4). This may be due to different dietary patterns between Japan and Singapore since free thiamine and TMP levels in plasma are sensitive to recent intake. Thiamine status, instead, is indicated by erythrocyte TDP or the functional assessment of erythrocyte transketolase (ETK) activity [41]. Nevertheless, consensus about case definitions for thiamine deficiency has not been achieved and various thresholds had been used in previous studies [41]. High intakes of caffeine, for example from coffee and tea, also interfere with thiamine absorption [43]. Given that high-carbohydrate diets and consumption of sugar-sweetened beverages, coffee, and tea is popular in Singapore [44], demand for TDP may be generally high and thiamine absorption may be suboptimal in the population. This may lead to the functionally suboptimal status of thiamine. On the other hand, given that free thiamine is phosphorylated into TDP once absorbed, a higher preconception free thiamine may be a result of suboptimal phosphorylation inhibiting the synthesis of TDP [45]. In both cases, our observation may suggest that functionally suboptimal TDP is associated with internalizing problems and pervasive developmental problems. ETK activity coefficient is computed as the ratio of stimulated ETK activity to basal ETK activity and indicates the availability of TDP [46]. However, ETK activity coefficient is not available in our study. Further investigation focusing on the functional suboptimal status of TDP is warranted.

In our exploratory analysis with brain MRI measures, thiamine was inversely associated with neonatal right subthalamic nucleus ODI, while neonatal right subthalamic nucleus ODI was inversely associated with multiple CBCL scores. Subthalamic hypo-activity has been reported in children with ASD symptoms [47]. This suggests that a potential mediating effect via neonatal ODI may exist for the positive associations of thiamine and internalizing problems and pervasive developmental problems, both of which are related to autism [48, 49]. Formal mediation analyses found that a small amount of the total effect of preconception thiamine on CBCL total problems were mediated by right subthalamic nucleus ODI (Supplementary Table 15). Due to the smaller sample sizes, these estimates were imprecise and did not pass our threshold for multiple testing.

The different timing of sample collection for biomarker measurement and behavioural symptom assessment may contribute to differences between our findings and those from previous studies. No previous study has looked at preconception measures, which may be critical to neurodevelopment. Due to large changes in plasma volume expansion and metabolism, concentrations measured during pregnancy may not reflect periconceptional levels. Previous studies have reported that lower maternal folate level during early pregnancy (<18 weeks gestation) was associated with childhood hyperactivity and peer problems [50] and with CBCL internalizing but not externalizing at age ~3 years [51]. In our analysis, Biomarker Cluster 4 (betaine, cobalamin, folate, trimethylamine N-oxide, vitamin D3) was suggestively associated with internalizing problems (Cluster PIP = 0.529) but none of the biomarkers in the cluster had been selected in more than 50% of the models (Conditional PIP < 0.5). This may suggest a critical window for folate during pregnancy rather than preconception.

Maternal supplement use and dietary patterns are usually assessed using questionnaires in epidemiologic studies [52, 53]. Such assessment is vulnerable to recall bias and social-desirability bias [54]. In addition, questions about the frequency of supplement use are often based on broad definitions, such as multivitamins [53]. Thus, misclassification may occur and precise intake could not be estimated. Objective measures of circulating micronutrients or metabolites are almost exclusively studied during pregnancy and not preconception itself [20]. During the very early stage of pregnancy, the embryo gets nutrients from fluids in the reproductive tract and endometrium [55, 56]. Thus, maternal circulating preconception biomarkers could indicate not only mother’s intake and metabolism of dietary constituents [57], but also fetal nutrient availability at conception and very early stage of pregnancy [58]. Thus in our study, investigation of circulating preconception biomarkers could be used as an indicator for both maternal nutritional status and fetal nutrient availability [57, 58], and may provide insight into the critical window for dietary interventions. This is not studied in most birth cohorts where participants were usually enroled either sometime after conception or at birth. In addition, existing studies on maternal biomarkers and offspring health outcomes mostly interrogated biomarkers individually or as a latent factor of multiple biomarkers [59, 60]. However, nutrients and metabolites do not act independently. We accounted for the complex interactions between biomarkers by applying K-means clustering and BKMR, which takes advantage of the kernel machine to characterise the exposure profile of multiple biomarkers and incorporate a Bayesian variable selection. This helps unravel the relevance of dietary constituents and potential biological pathway, as well as improve statistical power. Nevertheless, several limitations in our study should also be noted.

First, analyses in this study had small sample sizes and a relatively large number of biomarkers, which could decrease the efficiency of MCMC resampling. Therefore, we only consider findings with an MCMC effective sample size greater than 100 and a potential scale reduction factor smaller than 1.1. We also performed multiple additional analyses to evaluate the consistency of our findings. Second, BKMR analysis was not available for some subgroups in the stratified analyses due to the limited sample size. For those subgroups where both BKMR and linear regression were performed, larger Cluster and Conditional PIPs from BKMR corresponded to a smaller P-value from linear regression. Nevertheless, these results should be interpreted with caution. Third, sex-specific effects and critical windows may exist; however, we are not able to investigate this given the small sample size. Fourth, measurements of the erythrocyte TDP or functional assay of biological activity of thiamine (i.e., ETK activity) were not available. These could help examine if our conjecture of the functionally suboptimal status of TDP is true. Nevertheless, our findings suggest that monitoring functional biomarkers of thiamine may be more informative. Last but not least, child behavioural symptoms based on a parent-reported questionnaire may be biased by factors associated with a parent’s background. However, evaluation by investigators or clinicians is limited by the time they could spend on each child, thus it may not capture the daily behaviours of the child.

## Conclusion

In this study, we addressed a critical gap in the existing literature by investigating the associations of maternal circulating preconception biomarkers with parent-reported child behaviours to circumvent the confounding by pregnancy changes. By using clustering and mixture methods to account for the complex interaction between biomarkers, we identified associations of higher maternal preconception plasma thiamine and TMP with internalizing problems and pervasive developmental problems, suggesting that functional thiamine metabolism could be important in women planning for pregnancy. We further formally evaluated the extent to which neonatal brain microstructure mediated observed relationships. We did not find precise evidence for mediation via neonatal ODI at our given thresholds, further studies investigating other potential mediating mechanisms are needed.

## Supplementary information

Supplementary Figure

Supplementary Table

## Data Availability

Deidentified participant data will be made available on request.

## Code availability

The code is available upon request.
