# Supplementary material for: Maternal preconception circulating blood biomarker mixtures, child behavioural symptom scores and the potential mediating role of neonatal brain microstructure: the S-PRESTO cohort
Source: Transl Psychiatry. 2023 Feb 3;13:38. doi: 10.1038/s41398-023-02332-6 (PMC9898508; doi:10.1038/s41398-023-02332-6)
Supplement: Supplementary file 1 — Supplementary Figure [file 41398_2023_2332_MOESM1_ESM.docx]

**Maternal preconception circulating blood biomarker mixtures, child behavioural symptom scores and the potential mediating role of neonatal brain microstructure: the S-PRESTO cohort**

**Supplementary Figure**

**Figure 1** Exposure-outcome relationships using Bayesian kernel machine regression (BKMR) simultaneously accounting for 67 biomarkers (11 clusters), stratified by household income (Model 1, N=89 for lower household income group and N=110 for higher household income group). Biomarker levels and CBCL scores were in standard deviation unit. Grey areas indicate 95% credible interval.

**Figure 2** Associations of preconception thiamine and neonatal orientation dispersion index at 49 brain structures (n=56).

**Figure 3** Associations of preconception thiamine monophosphate and neonatal orientation dispersion index at 49 brain structures (n=56).

**Figure 4** Regression-based causal mediation analysis for preconception thiamine 🡪 neonatal ODI at the right subthalamic nucleus 🡪 CBCL scores (Child Behavior Checklist (CBCL), orientation dispersion index (ODI), n=62).


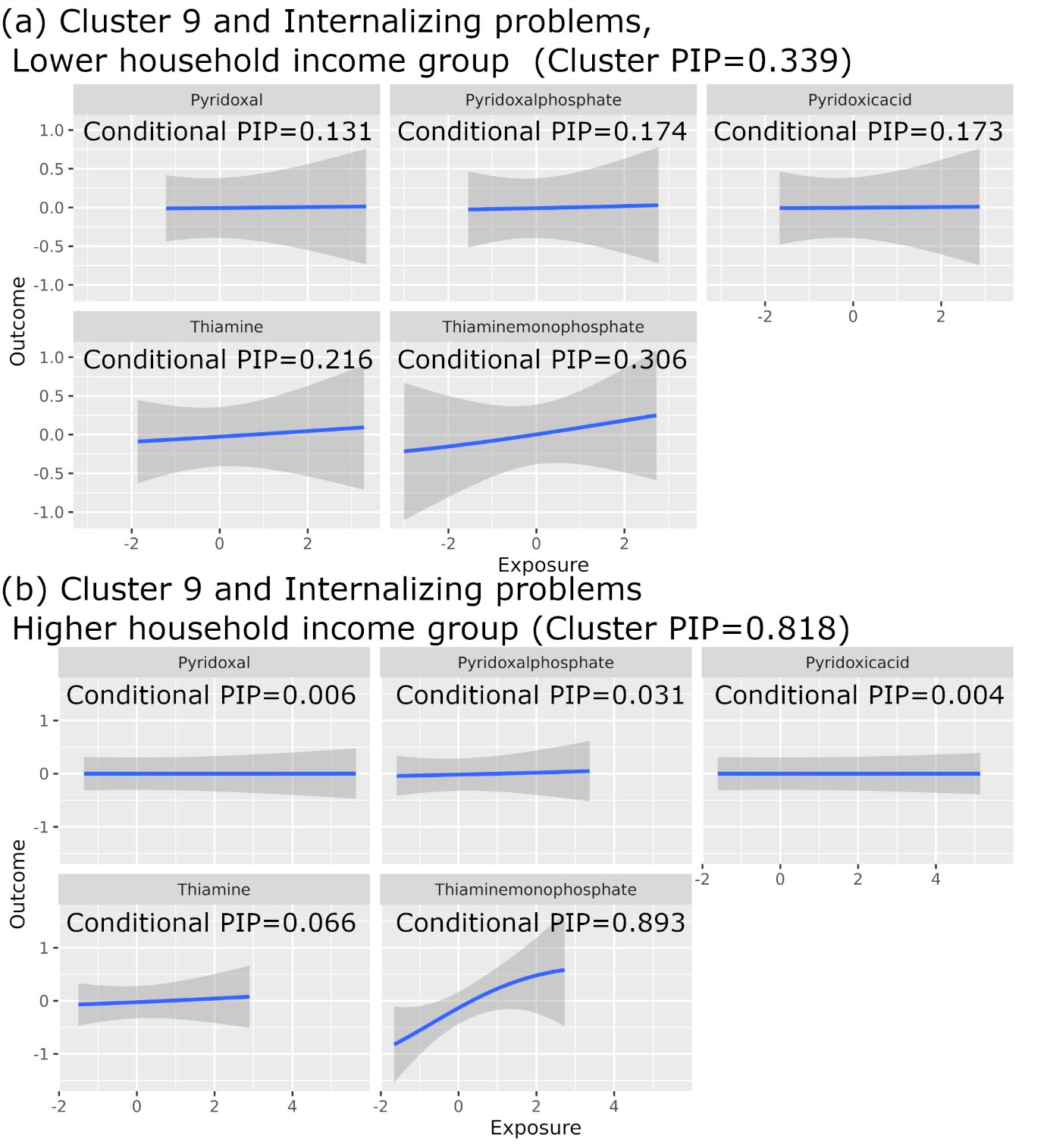


Figure 1 Exposure-outcome relationships using Bayesian kernel machine regression (BKMR) simultaneously accounting for 67 biomarkers (11 clusters), stratified by household income (Model 1, N=89 for lower household income group and N=110 for higher household income group). Biomarker levels and CBCL scores were in standard deviation unit. Grey areas indicate 95% credible interval.


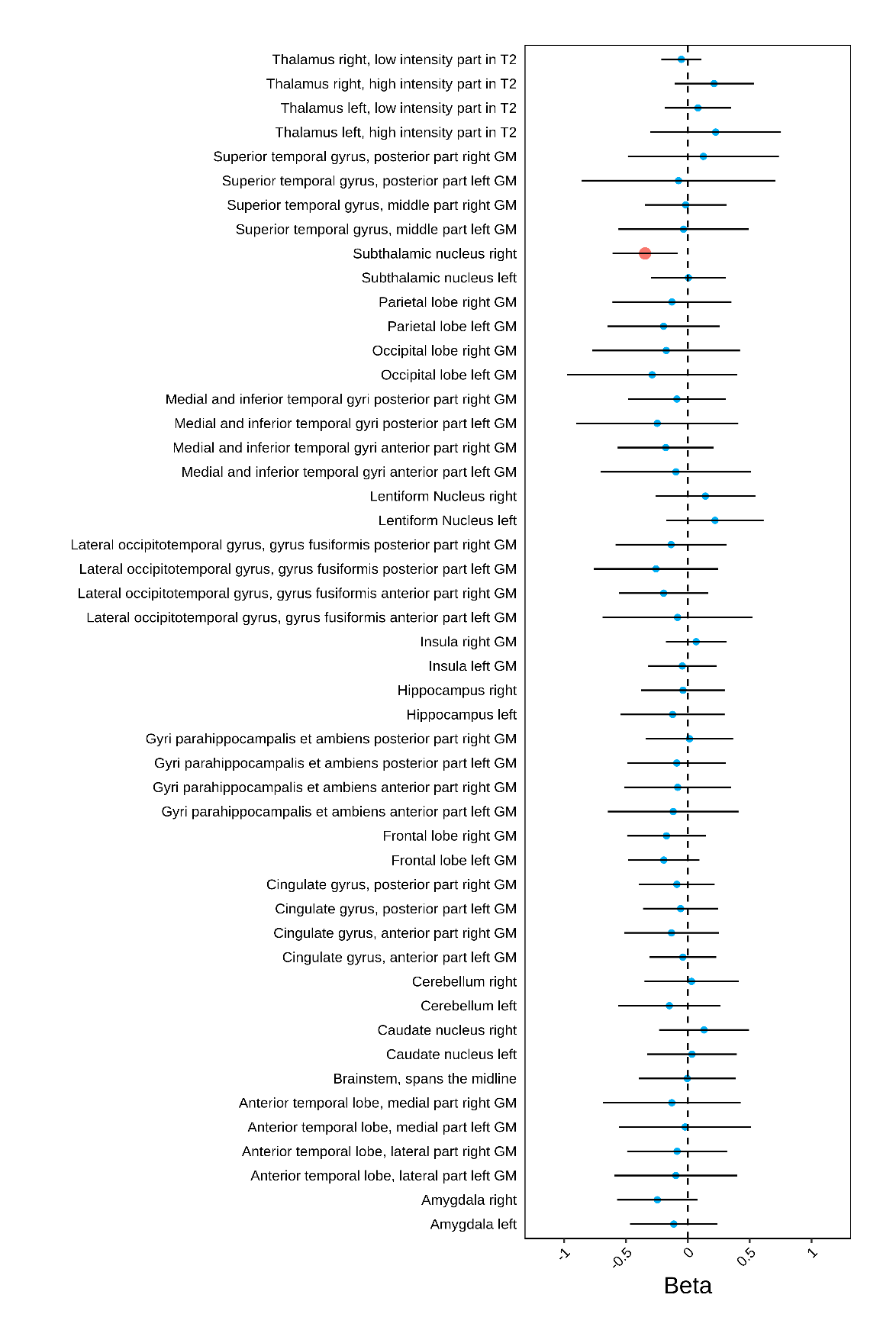


Figure 2 Associations of preconception thiamine and neonatal orientation dispersion index at 49 brain structures (n=56).


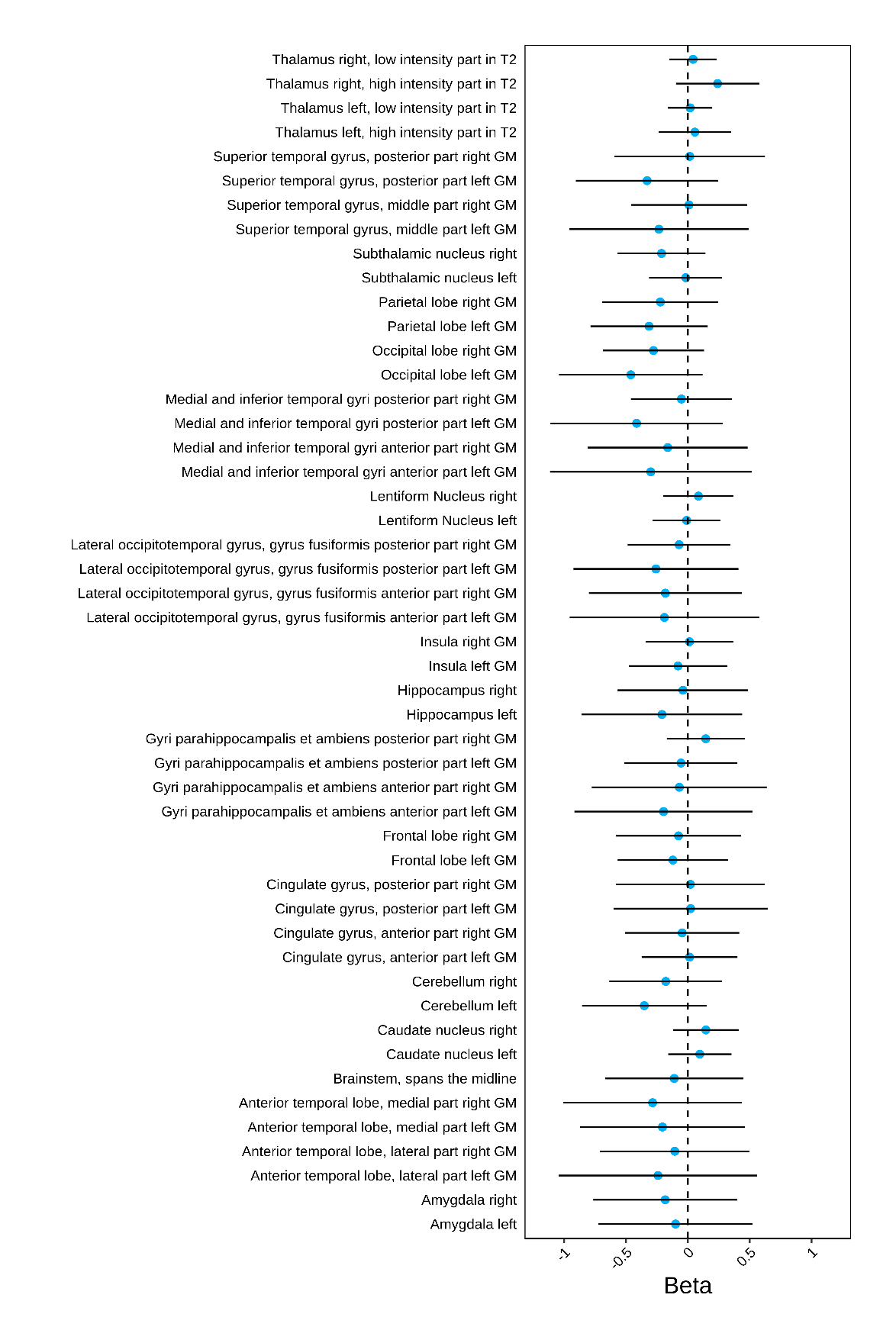


Figure 3 Associations of preconception thiamine monophosphate and neonatal orientation dispersion index at 49 brain structures (n=56).


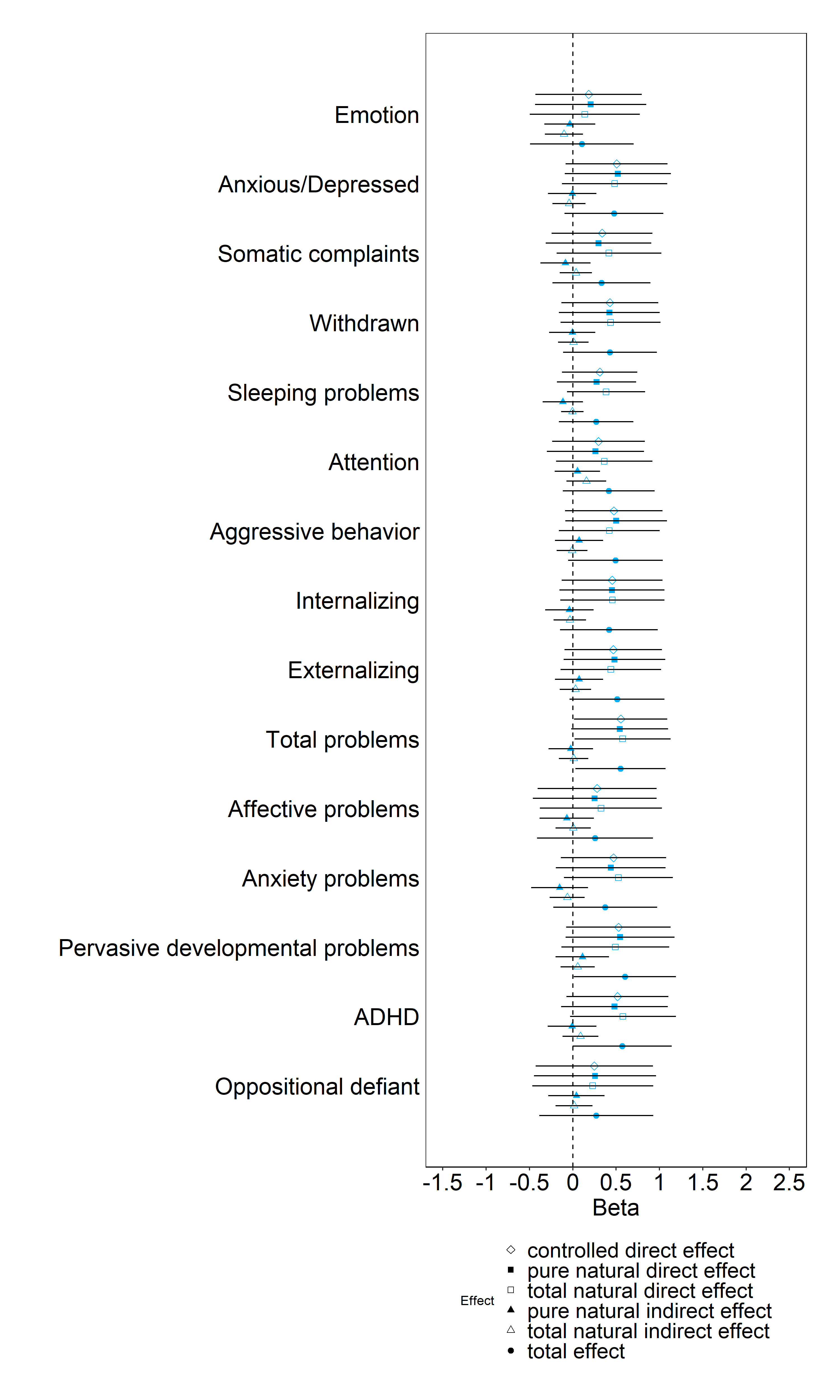


Figure 4 Regression-based causal mediation analysis for preconception thiamine 🡪 neonatal ODI at the right subthalamic nucleus 🡪 CBCL scores (Child Behavior Checklist (CBCL), orientation dispersion index (ODI), n=62).
